# Myanmarese Neuropathy: Clinical Description of Acute Peripheral Neuropathy Detected among Myanmarese Refugees in Malaysia

**DOI:** 10.1155/2014/187823

**Published:** 2014-10-28

**Authors:** Hiew Fu Liong, Datuk Puvanarajah Santhi, Viswanathan Shanthi, Rafia Mohd Hanip

**Affiliations:** Neurology Department, Kuala Lumpur Hospital, Jalan Pahang, 50586 Kuala Lumpur, Malaysia

## Abstract

*Background*. Since 2008, we have observed an increasing number of Myanmarese refugees in Malaysia being admitted for acute/subacute onset peripheral neuropathy. Most of them had a preceding history of starvation. *Methods*. We retrospectively studied the clinical features of all Myanmarese patients admitted with peripheral neuropathy from September 2008 to January 2014. *Results*. A total of 24 patients from the Chin, Rohingya, and Rakhine ethnicities (mean age, 23.8 years; male, 96%) had symmetrical, ascending areflexic weakness with at least one additional presenting symptom of fever, lower limb swelling, vomiting, abdominal pain, or difficulty in breathing. Twenty (83.3%) had sensory symptoms. Ten (41.6%) had cranial nerve involvement. Nineteen patients had cerebrospinal fluid examinations but none with evidence of albuminocytological dissociation. Neurophysiological assessment revealed axonal polyneuropathy, predominantly a motor-sensory subtype. Folate and vitamin B_12_ deficiencies were detected in 31.5% of them. These findings suggested the presence of a polyneuropathy related to nutrition against a backdrop of other possible environmental factors such as infections, metabolic disorders, or exposure to unknown toxin. Supportive treatment with appropriate vitamins supplementation improved functional outcome in most patients. *Conclusion*. We report a spectrum of acquired reversible neurological manifestations among Myanmarese refugees likely to be multifactorial with micronutrient deficiencies playing an important role in the pathogenesis.

## 1. Introduction

In Malaysia, where many Myanmarese people have been seeking refuge under the shelter of UNHCR, we observed over the past 6 years an increasing number of these refugees admitted with acute peripheral neuropathy. Most of the patients appeared malnourished with a preceding history of starvation of two to four weeks before onset of symptoms. These neuropathies manifested as acute to subacute onset, ascending, areflexic paralysis, with some having sensory and cranial nerve involvement. Electrophysiology studies showed axonal polyneuropathy and cerebrospinal fluid (CSF) analysis was usually acellular with normal protein levels.

In many instances, Guillain-Barré syndrome was initially suspected, but clinical and other biochemical features suggested that this was a different disease entity. Several therapeutic managements over time had been attempted with variable outcomes seen. Most were treated with various vitamin supplementations while some were given immunotherapy, for example, intravenous immunoglobulin infusion, despite lack of clinical and biochemical evidence of immune-mediated cause of peripheral neuropathy (Table 6).

However, little was known about the underlying etiology that led to these neuropathies and determining the cause remained a challenge, especially in the setting of rapidly progressing weakness though many of these acquired neuropathies were found to be reversible. We hypothesized that these acquired, axonal motor-sensory polyneuropathies could be related to multiple factors, such as nutritional deficiency, metabolic derangement, infections, or exposure to unknown toxins during their journey to Malaysia.

To understand the underlying etiology and pathogenesis of these neuropathies, we attempted to document the clinical spectrum with the biochemical and electrophysiology features of all the Myanmarese patients with acquired peripheral neuropathies at our centre.

## 2. Aims and Objectives

The primary objective of this review was to characterize the clinical features of the acquired peripheral neuropathies detected among Myanmarese patients admitted to the neurology ward at Kuala Lumpur Hospital. Electrophysiology studies were analyzed to determine the underlying pathological process of these neuropathies. The secondary objective was to identify the possible contributing factors leading to the onset of these peripheral neuropathies with available demographic data.

## 3. Material and Methods

This was a retrospective descriptive review of all Myanmarese refugees admitted with acute to subacute onset peripheral neuropathy from September 2008 to January 2014. Study population was Myanmarese refugees of both sexes and age above 12 who met the clinical case definition of peripheral neuropathy. Patients with existing underlying peripheral neuropathy of a known etiology were excluded. Myanmarese patients who were not refugees but had entered the country with valid documents were also excluded.

Demographic and clinical data from the admission records were reviewed. All presenting symptoms, neurological signs, nutritional parameters, cerebrospinal fluid analysis, and electrophysiology results leading to the diagnosis of peripheral neuropathy were documented. Pathological process was classified into either demyelinating or axonal subtype based on their electrophysiology features. In this review, functional disability was measured using disability scale of Hughes and colleagues (Hughes et al., 1978) [1], which consists of a simple 7-point scale (Table 1). Patients who could not walk independently had a score of grade 3 or more. This scale was used as a functional grading scale as many of the Myanmarese patients were treated as immune-mediated Guillain-Barré syndrome upon admission. The treatment administered was also analysed to evaluate the treatment outcome.

## 4. Results

### 4.1. Patients' Characteristics

**Figure 1:**
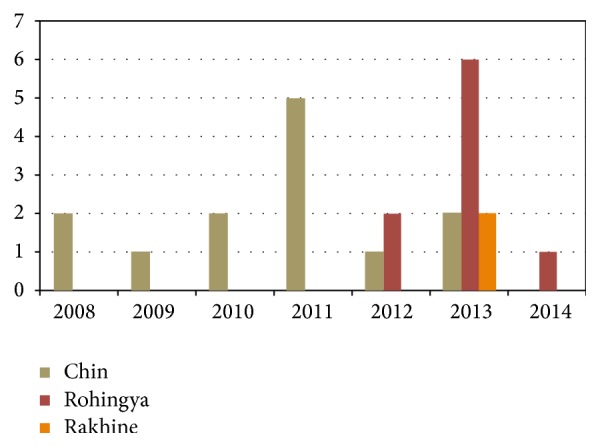
Number of cases of Myanmarese patients admitted to Hospital Kuala Lumpur from September 2008 to January 2014 (*n* = 24).

From September 2008 to January 2014, a total of 24 Myanmarese patients were hospitalised with ascending, areflexic weakness (Figure 1). Demographically, during the third quarter of 2008 till the middle of year 2012, the first 11 cases admitted were from the ethnic group Chin. Following the admission of the first Rohingya patient in 2012, the majority of the subsequent cases detected were predominantly Rohingya and Rakhine Muslims.

The majority of these patients were young adult males (96%) with a mean age of 23.8 years (range, 19–34 years). The Chin (54.2%) and Rohingya (37.5%) ethnicities made up the majority of the patients. The duration of their resettlement in this country ranged from four days to three years. The mean time from arrival in Malaysia to hospital admission amongst Rohingya Myanmarese patients was 19.1 days whereas ethnic Chin and Rakhine Myanmarese patients presented later with an average of 187.7 and 115 days, respectively, after resettling in Malaysia (Table 2).

No data prior to 2012 was available regarding the traveling and dietary history of these Myanmarese patients. However, eight Rohingya and two Rakhine Muslim Myanmarese patients admitted in the second half of 2012 were interviewed for details of their voyages from Myanmar to Malaysia. All of them had fled their country by sea in overcrowded fishing boats with a very restricted diet comprising small quantity (few spoonfuls) of water, plain white rice, dried fish, and egg, either once or twice per day. The boat journey was an average of 19.1 days (range 8–45 days) and upon arrival in a neighbouring country, they alleged they were often held captive in confined spaces with other survivors for a duration that ranged from 12 to 140 days (mean of 48.9 days) while awaiting deportation to Malaysia. The total duration of the journey from Myanmar to Malaysia was an average of 66.7 days (14–100 days).

### 4.2. Clinical Features

Patients presented to the hospital anywhere from seven days to 60 days (mean 27.75 days) after the onset of weakness. Clinically, at presentation, all of them had symmetrical, ascending areflexic weakness of the lower limbs, with 21/24 (87.5%) having upper limb involvement (Table 3). Sensory symptoms over the limbs were noted in 20 patients (83.3%). Cranial nerve involvement was noted in ten patients (41.6%). Facial weakness was present in nine patients (37.5%), bulbar weakness in five patients (20.8%), urinary retention in four patients (16.7%), nystagmus in two patients (8.3%), and extraocular weakness in one patient (4.2%). Confusion was noted in three patients (12.5%), who had prominent brainstem signs including bulbar weakness, nystagmus, and extraocular weakness. The mean time from onset of weakness to maximum disability was 22.9 days (range 5 to 60 days).

Antecedent events prior to the onset of limb weakness were noted in 13 patients (54.1%) with at least one of the following symptoms: fever (8/24; 33%), lower limb swelling (9/24; 37.5%), vomiting (7/24; 29.1%), abdominal pain (5/24; 20.8%), paresthesia (4/24; 16.7%), difficulty in breathing (3/24; 12.5%), and chest pain (1/24; 4.2%). None of them presented with preceding symptoms of diarrhoea. Two of the three patients with difficulty in breathing developed respiratory distress requiring mechanical ventilation and intensive care. The only female patient admitted was 30-week pregnant. All patients were young adults with no prior major medical problems such as diabetes mellitus, connective tissue diseases, and history of illicit drug usage/addiction. Two patients from ethnic Chin had an additional history of alcohol consumption on social occasions. None of them had any past history of bariatric or other gastrointestinal surgery.

### 4.3. Laboratory Investigations

For all 24 patients, total white cells and platelet counts on admission were normal. Two patients (8.3%) who had normocytic anaemia had concomitant* Plasmodium vivax malaria* (Malaria blood film diagnosis) and* Salmonella Typhi* infection (positive blood culture), respectively (Table 4). No macrocytosis (MCV > 100 fl) was detected in any Myanmarese patients tested and the average mean corpuscular volume (MCV) was 84.45 fl. Creatinine levels were elevated in 2/24 cases (8.3%) but not associated with elevated high blood urea nitrogen. Mean corrected serum calcium (2.2 mmol/L), magnesium (1.2 mmol/L), and phosphate (0.9 mmol/L) were within normal limits. Liver enzymes (alanine aminotransferase) were elevated (defined as >65 U/L) in 10/24 (41.6%) of the patients. Albumin levels were low (defined as <34 g/L) in five (20.8%) patients with average of 37.92 g/L. Sixteen patients were screened for human immunodeficiency virus and none of them showed reactive results. Only one of the 19 patients with viral hepatitis screening was positive for hepatitis B surface antigen testing with elevated liver enzymes while one other patient had detectable anti-hepatitis C antibody but liver function test was within normal range. Four patients were screened for serum cytomegalovirus IgM level and IgM herpes simplex virus 1 and 2 serology was sent for six other patients but all were reported negative. Thyroid function was tested in thirteen patients and was reported as within normal limits. Vitamin level studies were available in 19 of 24 patients (79.2%). Six (35.3%) of the 17 patients were found to be deficient for folic acid (plasma folate < 4.5 nmol/L). Among these patients, one had been in Malaysia for over 6 months. Seven (36.8%) of the 19 patients with serum vitamin B12 levels had normal levels (>350 pg/mL). Six (31.5%) patients had low B12 levels (<200 pg/mL) and another six (31.5%) had borderline low levels (201–350 pg/mL). One Rohingya patient with low folate and vitamin B12 level also demonstrated elevated homocysteine level of 19.35 *μ*mol/L (5.08–15.39). Serum lead was normal in one patient (0.22 umol/L, Normal: <0.7 umol/L).

Cerebrospinal fluid (CSF) analysis was carried out in 18 patients (75%) after at least 7 days from symptom onset. All the CSF studied showed no evidence of albumin-cytological dissociation. The mean CSF protein was 0.259 g/L (range 0.13–0.41 g/L) while the mean CSF glucose was 3.75 mmol/L (range 3.1–4 mmol/L). Of these 18 patients, two of them had positive cell count of polymorphs, five and 40 cells/mL respectively, with normal CSF protein level. One patient who developed respiratory distress and required invasive ventilation had an antiganglioside antibody that was negative.

Neurophysiology examinations were carried out in all patients within 7 days of admission. The mean duration of nerve conduction study performed from the onset of limb weakness was 24.5 days (range: day 9 to day 57). All of them had primary axonal polyneuropathy, with 19/24 (79.2%) predominantly motor-sensory subtype and 5/24 (20.8%) patients with pure motor neuropathy. Among the 19 patients with motor-sensory axonal subtype, three Rohingya and one Chin Myanmarese patients had inexcitable nerve studies. No evidence of primary demyelination was seen in the electrophysiology examinations and all F-waves were within the normal limits with the exception of the three patients with inexcitable nerve studies.

### 4.4. Clinical Outcome

At presentation, the functional classification based on Hughes' functional scale was as follows: two patients (8.4%) were grade 5, 12 patients (50%) were grade 4, and ten patients (41.6%) were grade 3 (Table 5). Two (8.3%) of the patients had respiratory distress requiring invasive ventilation support and intensive care. None of them died during their stay in the hospital. All three patients with confusion recovered full consciousness with no cognitive disabilities. Ethnic group Rohingya patients were more severely affected in terms of functional disability with 89% presenting with functional classes 4 and 5. Chin and Rakhine Myanmarese patients presented with less disability (functional classes 4 and 3).

Eight patients (33.3%) were treated with intravenous immunoglobulin (IVIg) infusion, followed by vitamin B, folate, and multivitamin supplementation. Sixteen (66.7%) were treated conservatively with only a combination of vitamins B, folate, and multivitamins. Vitamin B supplementations were in the form of thiamine (B1), neurobion (B1, B6, and B12), Methylcobalamin (B12), and B complex.

The mean duration of hospital stay was 17.04 days (range: 1–57 days). Twenty (83.3%) patients were discharged home and the remaining four (16.7%) were transferred to a rehabilitation ward (*3 Rohingya patients and 1 Chin Myanmarese patient*). None of the patients succumbed to the disease. Five of the eight (62.5%) patients given intravenous immunoglobulin showed a functional improvement score of one, within 60 days after admission. Three patients (37.5%) had mild-to-moderate disability with a functional scale of 3 or better. Among the patients treated with vitamin supplementation, seven of 16 (43.8%) patients showed a functional improvement score of at least one, within 60 days. Thirteen patients (81.3%) had mild-to-moderate disability with functional scale of 3 or better. All three patients with inexcitable nerves studies had poor functional disability score of 4 on admission and showed no functional recovery after 60 days.

## 5. Discussion

This retrospective review documents a spectrum of neurological manifestations among Myanmarese refugees in Malaysia in association with a preceding, severely restricted diet, following a rough journey from Myanmar. The presentations ranged from motor-sensory peripheral neuropathy to a compromised cardiovascular system manifested as chest pain, shortness of breath, and bilateral lower limb swelling. Some had also confusion, nystagmus, and ophthalmoplegia that was suggestive of encephalopathy (central nervous system involvement). Electrophysiology studies showed motor-sensory or pure motor, axonal polyneuropathy. However, CSF analysis after the first week showed normal biochemistry without evidence of albumin-cytological dissociation. Recovery in functional disability was seen in patients treated with intravenous immunoglobulin and multivitamins supplementation for both groups.

The initial appearance of these neurological manifestations presented a diagnostic quandary in that these patients were often diagnosed to have immune-mediated Guillain-Barré syndrome (GBS) with some even having received intravenous immunoglobulin therapy. Unlike the classical cases of Guillain-Barré syndrome (GBS), the atypical nature of the clinical presentations, electrophysiological and cerebrospinal fluid studies led to the consideration of other acquired external factors as the possible etiology. Mainly, Myanmarese male patients from the three subethnic populations of Rohingya, Rakhine Muslim, and Chin were affected.

Limited data is available on the number of Myanmarese refugees in Malaysia with neurological conditions. In the past, outbreaks of neuropathic conditions associated with micronutrient deficiencies have occurred in many parts of Asia beginning in the 1960s. In 1966, Thanangkul and Whitaker reported 24% incidence of peripheral neuropathy secondary to thiamine deficiency among adults and adolescents in Chiang Rai Province [2]. From October 1993 to June 1994, 12,000 cases of peripheral neuropathy were detected among 85,000 Bhutanese refugees in Nepal and suspected to be dry beriberi [3]. More recently in 1999, a beriberi outbreak in Taiwan shows that at least 19% of Chinese refugees met the criteria for a probable case with 77% of them showing weakness of extremities [4]. In comparison to the previous epidemiology reports, we suspect that our figure of 24 patients from a single tertiary centre is underrepresentative of the actual number affected.

As of the end of December 2013, there were approximately 131,387 Myanmar refugees and asylum seekers registered with UNHCR in Malaysia. These were composed of 52,152 Chins, 32,611 Rohingya, 11,713 Myanmar Muslims, 7,940 Rakhine, and other ethnicities from Myanmar [5]. While 70% of refugees that arrive in Malaysia are men, our demographic data of predominant Myanmarese male (96%) demonstrates strong gender selectivity. At present, no conclusive explanation can be inferred from our small cohort of patients until a larger epidemiology study is carried out.

Initially, the disease was thought to be primarily an axonal loss in the peripheral nervous system giving rise to neuropathy. However, some cases exhibited multiple cranial nerves palsies with confusion, indicating that neuronal injury was also occurring in the central nervous system, particularly the brainstem. There are many possible etiologies that could be attributed to the above presentations. Environmental factors such as nutritional deficiency, metabolic and electrolyte disturbances, exposure to unknown toxins, infection and postinfection, or immune-related neuropathy could all lead to a similar spectrum of neurological presentation. The emergence of this clinical picture coincidentally resembled the neurological complications of thiamine deficiency following bariatric surgery among obese patients [6] and peripheral neuropathies among populations vulnerable to micronutrients' deficiencies, for example, prison population [7], detainees [4], and refugees [8].

Thiamine (vitamin B1) is a water-soluble vitamin that has limited body stores and individuals subjected to a thiamine-poor diet can deplete these stores within two weeks. Thiamine deficiency in chronic state results in a clinical syndrome manifested as anorexia, malaise, and weakness of the legs, frequently associated with paresthesia. Limb oedema and palpitation may coexist. This chronic state may, at any time, progress to an acute condition characterised either by cardiac involvement with peripheral oedema or acute peripheral neuropathy [9]. In a more severe case, Wernicke's encephalopathy may develop as a result of biochemical lesions in the central nervous system, leading to confusion, ataxia, and ophthalmoplegia. However, we are unable to determine the serum thiamine levels or RBC transketolase activity among the above group of Myanmarese refugees due to unavailability of laboratory support.

Based on our findings, ethnic Rohingya males have a higher susceptibility to a more severe neurological manifestation (disability) of peripheral neuropathy. The reason why this happens in this subgroup remains unclear. Rohingya refugees come from the state of Rakhine with background of high prevalence in Global Acute Malnutrition (GAM) index, above the WHO emergency threshold of 15% (GAM 2008 = 22.7%, GAM 2009 = 20.2%) [10]. Political upheaval in both the Chin and Rakhine minority states in Myanmar's western border has seen massive population displacement to other countries in the region [11, 12]. Various environmental factors throughout the journey could increase the risk of thiamine depletion among this group of population. As thiamine is essential in the metabolism of carbohydrates to liberate energy, the daily requirements of thiamine increase in many conditions, for example, intercurrent febrile illnesses during their journey (Malaria and typhoid infection). Physical stress, starvation, hyperthyroidism, pregnancy, and lactation could all lead to further worsening of thiamine depletion, resulting in various thiamine-related neurological manifestations.

The diagnosis of nutritional neuropathy, recognized initially to be associated with vitamin B1 deficiency, is often not as straightforward as many clinicians presume. In the past, similar conditions have been reported where single thiamine hypovitaminosis was thought to be the cause. In 1992-1993, as a result of food shortage in Cuba, widespread thiamine depletion led to an epidemic of optic and peripheral neuropathy (*Cuban neuropathy*) involving more than 50,000 Cubans [13]. However, only 30–70% of those with neuropathy demonstrated biochemical evidence of abnormal thiamine status. It is likely that depletion of thiamine among Cubans at that point also reflected concomitant depletion of many other nutrients. This serves to illustrate the complexity of human malnutrition [14].

From the above group of Myanmarese refugees with peripheral neuropathies, not all presented with severe nutritional deficiency parameters as evidenced by the presence of anemia in only 2/24 (8.3%) and low albumin in 4/24 (16.7%) patients. Folate deficiency was noted in one-third of the patients and vitamin B12 deficiency in two-thirds of them despite none with elevated mean corpuscular volume (MCV). Serum electrolytes including corrected calcium, phosphate, and magnesium levels were all within normal limits. Folate and vitamin B_12_ deficiency in particular increases the risk of peripheral neuropathy [15, 16]. Neurological manifestations of vitamin B_12_ deficiency include myelopathy and peripheral neuropathy, autonomic and optic [16]. The B_12_ deficient peripheral neuropathy is mainly sensory and of axonal pathology [17]. Early diagnosis is critical because neuropathies due to folate and cobalamin deficiencies are potentially reversible [17, 18]. In adults, total body cobalamin content is 2–5 mg. A complete discontinuation in cobalamin absorption will take 3–5 years to deplete cobalamin stores [19]. Among our group of Myanmarese patients with low and borderline low serum vitamin B_12_ level, 20% of them had been already residing in Malaysia for more than six months. This indicates the chronicity of the background nutritional deficiency status and the complexity of vitamin B_12_ deficiency state.

Another interesting point to highlight from the results is that many of these Myanmarese patients with peripheral neuropathy had also cranial nerves' manifestation. The combination of generalized areflexic flaccid paraparesis with facial diplegia, ophthalmoplegia, and bulbar weakness often leads to the diagnosis of acute polyradiculoneuropathy (GBS) or its variant. GBS could have been mimicked by dry beriberi due to thiamine deficiency [20, 21]. The recent onset of areflexic flaccid paralysis would make the diagnosis of GBS a sensible one, especially with electrophysiological studies demonstrating motor-sensory, axonal polyneuropathy. However, CSF studies after the first week of symptoms did not reveal evidence of albumin-cytological dissociation making this group of peripheral disorders highly atypical for GBS. Although some of the patients began by mimicking acute onset GBS, its variable clinical course and CSF findings were not supportive. Unfortunately, antiganglioside antibodies were only available in one patient with recent onset acute flaccid paralysis and respiratory distress requiring mechanical ventilation and the test was negative. Viral screening was only carried out in a small number of patients and should not be construed as ruling out possible viral infection with the above presentation. Furthermore, the lack of cellular response would rule against an infective cause for the axonal neuropathy.

In terms of neurological and functional improvement, the majority of the patients (21/24; 87.5%), irrespective of treatment with intravenous immunoglobulin (IVIg) plus multivitamins or multivitamins alone, showed a functional improvement score of at least 1 within 60 days with the exception of three patients with inexcitable nerves on electrophysiology studies. No fatality case was noted. When all is said and done, no deductions can be made as to whether neurological improvement resulted from IVIg administration, multivitamins supplementation, or the natural evolution of the disease. However, the effort of supplementing supraphysiological doses of various vitamins with or without immunotherapy did not appear to work immediately or significantly well. This clearly highlights the importance of continuous research effort in identifying the actual cause of the disease and prevention.

The main factors that appeared to affect the functional recovery in our patients were the duration of prolonged restricted food supply, the severity of functional impairment at presentation (Hughes' functional scale ≥4), and the degree of axonal loss on electrophysiology studies. Other indicators of poor functional recovery were the presence of concomitant stress factors particularly infection, central nervous system involvement, and being an ethnic Rohingya.

There were limitations in our study. Firstly, we reviewed only Myanmarese refugees admitted to one tertiary center in Kuala Lumpur. Hence, our sample size is probably underrepresentative as similar cases have since been reported in hospitals around Malaysia. Secondly, clinical and biochemical parameters were not prospectively collected, limiting the completeness of our data while laboratory biochemical diagnosis was also limited. We hope in the future to include other diagnostic tests, for example, serum vitamin B1 levels, RBC transketolase activity, homocysteine, methylmalonic acid, and blood vitamin B6 levels. Thirdly, the neurology outcomes, in this group of patients, were not systematically examined using a standardised disability scale and scheduled follow-up. Finally, we also acknowledge that there may be a possibility of an underlying gender and genetic susceptibility especially seen among the Rohingya Myanmarese males. In the future, genetic studies, morphological characterization of peripheral nerve biopsies, and the long-term course of the disease will need to be further investigated.

## 6. Conclusion

Among Myanmarese refugees, we report the presence of a spectrum of acquired, reversible neurological manifestations ranging from common presentation of motor-sensory axonal polyneuropathy to a more severe but rare central nervous system involvement. We attributed this to be multifactorial with micronutrient deficiencies playing an important role in the pathogenesis. In light of this review, early health assessment for refugees after resettlement in a new host country is recommended to introduce early identification and timely intervention of supplements. This also highlights the need to raise awareness among the international community of the much-needed, long-term strategies to be adopted in populations worldwide, especially refugees, affected by emergencies.

## Figures and Tables

**Table 1:** Hughes' functional scale [1].

Scale	Function
0	Healthy
1	Minor symptoms or sign of neuropathy but capable of manual work/capable of running
2	Able to walk without support of a stick (5 m across an open space) but incapable of manual work/running
3	Able to walk with a stick, appliance, or support (5 m across an open space)
4	Confined to bed or chair bound
5	Required assisted ventilation (for any part of the day or night)
6	Death

**Table 2:** Demographic data of the patients.

Ethnic	*N* (%)	Mean age (years)	Gender (*n*)		Average duration in Malaysia (days)	Length of hospital stay (days)	Smoker	Alcohol
		Range 19–34 (mean = 24.04)	Male	Female	Range 4–365 (days)			
Chin	13 (54.2%)	24.8	12	1	187.7	9.4	0	2
Rohingya	9 (37.5%)	23.7	9	0	19.1	29.8	3	0
Rakhine Muslim	2 (8.3%)	27	2	0	115	9.5	1	0

**Table 3:** Characteristics clinical features of Myanmarese patients.

Symptoms	Number of patients *n* (%)	Chin (*n*)	Rohingya (*n*)	Rakhine Muslim (*n*)
Lower limb swelling	9/24 (37.5%)	3	5	1
Fever	8/24 (33.3%)	1	5	2
Vomiting	7/24 (29.1%)	3	4	0
Abdominal pain	5/24 (20.8%)	2	2	1
Paresthesia	4/24 (16.7%)	0	3	1
Shortness of breath	3/24 (12.5%)	1	2	0
Sore throat	3/24 (12.5%)	2	1	0
Chest pain	1/24 (4.2%)	0	0	1

Clinical features				

Weakness distribution				
(i) LL > UL	12/24 (50%)	5	6	1
(ii) LL = UL	9/24 (37.5%)	6	3	0
(iii) LL only	3/24 (12.5%)	2	0	1
Motor-sensory	20/24 (83.3%)	11	8	1
Pure motor	4/24 (16.7%)	2	1	1
Confusion	3/24 (12.5%)	0	3	0

Cranial nerves				
(i) Facial weakness	9/24 (37.5%)	2	7	0
(ii) Bulbar weakness	5/24 (20.8%)	1	4	0
(iii) Extraocular weakness	1/24 (4.2%)	0	1	0
Nystagmus	2/24 (8.3%)	0	2	0
Urinary retention	4/24 (16.7%)	0	4	0

Medical conditions				

(i) Malaria	1/24 (4.2%)	0	1	0
(ii) Salmonella Typhi	1/24 (4.2%)	0	1	0
(iii) Pregnancy	1/24 (4.2%)	1	0	0

UL: upper limbs; LL: lower limbs.

**Table 4:** The laboratory and electrophysiology features of Myanmarese patients.

Laboratory results	Number of patients *n* (%)	Chin (*n*)	Rohingya (*n*)	Rakhine Muslim (*n*)
Haemoglobin				
Low	2/24 (8.3%)	0	2	0

Mean corpuscular volume				
(i) Low	7/24 (29.1%)	5	1	1
(ii) Normal	17/24 (70.8%)	8	8	1

Liver function				
(i) Low albumin	4/24 (16.7%)	1	3	0
(ii) Elevated ALT	12/24 (50%)	2	8	2

Serum folate				
Low	6/17 (35.3%)	3/8 (37.5%)	3/7 (42.8%)	1/3 (33%)

Serum vitamin B12				
(i) Low	6/19 (31.5%)	3/9	3/8	0/2
(ii) Borderline low	6/19 (31.5%)	3/9	2/8	1/2

Electrophysiology				

Motor-sensory axonal polyneuropathy	19/24 (79.2%)	10	8	1
Pure motor axonal polyneuropathy	5/24 (20.8%)	3	1	1

**Table 5:** Hughes' functional scale classification of Myanmarese at presentation.

Ethnic	*N* (%)	Functional class 5	Functional class 4	Functional class 3
Chin	13 (54.2%)	0	6 (46%)	7 (54%)
Rohingya	9 (37.5%)	2 (22%)	6 (67%)	1 (11%)
Rakhine Muslim	2 (8.3%)	0	0	2 (100%)

Total	24 (100%)	2 (8.4%)	12 (50%)	10 (41.6%)

**Table 6:** The functional score on admission and within 60 days.

Ethnic	*N* (%)		IVIG + vitamins				
			Functional classification (*N*)				
			5	4	3	2	1
Chin	13 (54.2%)	Admission	—	2	1	—	—
		Within 60 days	—	1	1	1	—
Rohingya	9 (37.5%)	Admission	2	3	—	—	—
		Within 60 days	—	4	1	—	—
Rakhine Muslim	2 (8.3%)	Admission	—	—	—	—	—
		Within 60 days	—	—	—	—	—

Total	24 (100%)	Admission	2	5	1	—	—
		Within 60 days	—	5	2	1	—

Ethnic	*N* (%)		Vitamins				
			Functional classification				
			5	4	3	2	1

Chin	13 (54.2%)	Admission	—	4	6	—	—
		Within 60 days	—	2	6	2	—
Rohingya	9 (37.5%)	Admission	—	3	1	—	—
		Within 60 days	—	1	2	1	—
Rakhine Muslim	2 (8.3%)	Admission	—	—	2	—	—
		Within 60 days	—	—	1	—	1

Total	24 (100%)	Admission	—	7	9	—	—
		Within 60 days	—	3	9	3	1
